# Acupuncture modulates the microbiota-gut-brain axis to treat irritable bowel syndrome: a mechanistic exploration

**DOI:** 10.3389/fnins.2026.1820371

**Published:** 2026-06-10

**Authors:** Linhui Wu, Junyu Fang, Meilin Chen, Yuan-Cheng Si

**Affiliations:** College of Acupuncture and Tuina, Guizhou University of Traditional Chinese Medicine, Guiyang, Guizhou, China

**Keywords:** acupuncture, gut microbiota, irritable bowel syndrome, mechanism, microbiota-gut-brain axis

## Abstract

Irritable bowel syndrome (IBS) is a common functional gastrointestinal disorder involving dysregulation of the microbiota-gut-brain (MGB) axis. Acupuncture effectively alleviates IBS symptoms, yet its underlying mechanisms remain incompletely understood. This review synthesizes current evidence to propose a mechanistic framework by which acupuncture treats IBS through MGB axis modulation. We systematically examine: (1) MGB axis dysfunction in IBS pathophysiology across neural, endocrine, and immune pathways; (2) acupuncture’s modulation of gut microbiota structure (alpha/beta diversity, specific bacterial genera); (3) functional consequences including enhanced short-chain fatty acid production and tryptophan metabolism; (4) causal evidence from fecal microbiota transplantation; (5) correlations between microbiota changes and clinical improvement. Key findings reveal that acupuncture induces “convergent remodeling” of microbial structure toward a healthy profile, exerts “bidirectional regulation” on beneficial and harmful bacteria, and establishes a “niche selection” mechanism via neuro-immune pathways. These microbiota-mediated effects integrate with neural, endocrine, and immune pathways, forming a “point-to-surface” networked regulatory pattern that explains acupuncture’s dual efficacy in alleviating both gastrointestinal and psychological symptoms. This review provides a novel theoretical framework for understanding acupuncture’s therapeutic mechanisms and supports its clinical application in IBS management.

## 1 Introduction

Irritable bowel syndrome (IBS) is a functional bowel disorder diagnosed by excluding organic diseases. Its clinical features include abdominal discomfort and altered bowel habits (Koerner and Sullivan, 2020), with typical symptoms such as abdominal pain, diarrhea, and changes in stool frequency or form (Cuber et al., 2023). This condition is globally prevalent, with a worldwide prevalence of approximately 9.2% and marked regional variation ranging from 0.2 to 29.9% (Takeoka et al., 2023). Epidemiological studies have identified women and young adults as independent risk factors for the development of IBS (Mujamammi et al., 2023). In China, the prevalence of IBS is estimated to be between 5 and 12% (Li B. et al., 2020). Given its high prevalence, IBS is considered a major clinical challenge globally in gastroenterology.

To date, the exact pathophysiological mechanisms of IBS remain incompletely understood. However, dysfunction of the gut-brain axis (GBA) is considered a core mechanism. This concept provides a critical framework for understanding IBS pathophysiology and has been systematically integrated into the biopsychosocial model to guide diagnosis and treatment (Cashman et al., 2016). The GBA is a complex bidirectional communication network between the gut and the brain, involving neural, endocrine, and immune pathways (Vicentini et al., 2021). Accumulating evidence suggests that abdominal pain—often accompanied by diarrhea or altered bowel habits—is closely associated with dysregulation of this bidirectional communication pathway.

Within this complex system, the gut microbiota (GM) plays a critical role. Through its metabolites (e.g., short-chain fatty acids) and neuroactive substances, the gut microbiota interacts with the GBA, collectively forming the “microbiota-gut-brain axis” (MGB axis) (Hattori and Yamashiro, 2021). Specifically, gut microbiota dysbiosis can disrupt the integrity of the intestinal epithelial barrier, leading to increased permeability. This pathological change can further activate local immune responses and induce persistent low-grade inflammation (Forsythe and Kunze, 2013; Matteoli and Boeckxstaens, 2013). These locally originating pathophysiological signals can ascend to the central nervous system (CNS) via multiple pathways, including afferent vagal nerves. Concurrently, the CNS is not a passive recipient; it actively modulates intestinal immune function and cytokine balance through descending regulatory mechanisms, such as the cholinergic anti-inflammatory pathway mediated by efferent vagal nerves, thereby influencing the intestinal microenvironment in return (Pan et al., 2024). Thus, dysregulation of this bidirectional communication is considered a crucial basis for the generation and perpetuation of IBS symptoms.

Acupuncture is recognized as an effective therapeutic intervention for irritable bowel syndrome (IBS). Emerging evidence indicates that its effects may involve modulating the gut microbiota composition and restoring microecological balance. Although accumulating evidence shows that acupuncture can alter gut microbiota in IBS, it remains unclear which specific microbial metabolites or neuroactive pathways mediate its effects on visceral hypersensitivity. Moreover, the bidirectional nature of the MGB axis—whether acupuncture primarily acts peripherally (on the gut) or centrally (on the brain)—has not been critically evaluated.

Therefore, this review synthesizes current evidence to propose a mechanistic framework by which acupuncture modulates the MGB axis, focusing on the peripheral-to-central signaling pathways and the central-to-peripheral regulatory loops, to treat IBS.

## 2 Role of the microbiota-gut-brain axis in IBS pathophysiology

The pathogenesis of IBS is closely linked to dysregulation of the gut-brain axis, with the gut microbiota serving as key modulators that influence neural, endocrine, and immune pathways (Fülling et al., 2019; Hillestad et al., 2022). In IBS, gut microbiota dysbiosis is considered a key initiator of MGB axis disruption. This dysbiosis first compromises the integrity of the intestinal epithelial barrier, subsequently activating the intestinal immune system and leading to the release of pro-inflammatory cytokines. These locally generated changes exert a dual effect: they directly trigger abnormalities in intestinal motility and sensory function, while also acting as ascending signals transmitted to the central nervous system (CNS) via pathways such as the vagus nerve. This transmission activates the hypothalamic-pituitary-adrenal (HPA) axis, elevating stress hormones like cortisol. In turn, elevated circulating cortisol feeds back to the gut as a descending signal, further exacerbating intestinal permeability, inflammation, and visceral hypersensitivity—forming a self-reinforcing vicious cycle (Distrutti et al., 2016). Neuroimaging studies provide direct evidence for this framework. Functional magnetic resonance imaging (fMRI) has revealed significant correlations between gut microbiota composition and both the volume and functional connectivity of key brain regions (Labus et al., 2019), supporting the involvement of the MGB axis in IBS.

### 2.1 Neural pathways: from afferent signaling to central sensitization

Neural pathways constitute a critical component of bidirectional MGB axis communication. Microbial metabolites are transmitted to the CNS via two parallel sensory pathways—the vagal and spinal afferent pathways—with the vagal pathway being a key regulator of gastrointestinal homeostasis.

Under physiological conditions, SCFAs—produced by gut microbiota from dietary fiber—activate free fatty acid receptors on enterochromaffin (EC) cells, triggering the synthesis and release of serotonin (5-hydroxytryptamine, 5-HT). This 5-HT then binds to receptors (e.g., 5-HT_3_ and 5-HT_4_) on intrinsic primary afferent neurons, activating the afferent vagus nerve. Signals are transmitted to the nucleus tractus solitarius in the brainstem, enabling precise regulation of intestinal secretion, motility, and sensation (van Thiel et al., 2023; Alcaino et al., 2025).

However, in IBS, gut microbiota dysbiosis alters the SCFA profile, leading to excessive 5-HT release. Elevated serotonin activates 5-HT_3_ and 5-HT_4_ receptors, inducing hypercontractility and dysmotility. Concurrently, sustained high serotonin levels continuously activate dorsal root ganglion (DRG) neurons, significantly reducing their pain threshold—a core mechanism of visceral hypersensitivity (VHS) (van Thiel et al., 2023). This persistent afferent barrage may further drive central sensitization, enhancing pain perception at the spinal and supraspinal levels.

Beyond direct effects on serotonin release, the gut microbiota influences neurotransmitter balance by modulating tryptophan metabolism. Most of the body’s serotonin is synthesized in the gut; acupuncture-optimized microbiota can regulate tryptophan flux—either by stimulating EC cells via SCFAs to produce serotonin or by modulating bacterial enzyme activity (Jang et al., 2020; Zhang et al., 2025). Normalizing serotonin levels is critical for maintaining gut motility and visceral sensitivity; in IBS, acupuncture corrects aberrant serotonin release via this pathway, alleviating dysmotility and visceral hypersensitivity (Qi et al., 2025).

Furthermore, central serotonin deficiency is considered closely associated with the anxiety and depression commonly comorbid in IBS. Microbial metabolites play a critical regulatory role: SCFAs stimulate tryptophan hydroxylase 1 expression in EC cells, promoting serotonin synthesis; conversely, pro-inflammatory cytokines (e.g., IFN-γ) activate the indoleamine 2,3-dioxygenase pathway, depleting tryptophan and thus limiting serotonin synthesis. This imbalance between peripheral and central serotonergic systems is considered a key link between intestinal symptoms and psychiatric comorbidities (Fukumoto et al., 2003; Zoga et al., 2014; Reigstad et al., 2015; Wang L. et al., 2022).

In contrast to the vagal pathway—primarily responsible for physiological sensation—the spinal afferent pathway is the main route for transmitting nociceptive signals (e.g., pain). Sensory neurons in this pathway have their cell bodies in the dorsal root ganglia (DRG), with central axons projecting to the spinal dorsal horn to synapse with second-order neurons (Distrutti et al., 2016; Banfi et al., 2021; van Thiel, 2022). Peripheral sensitization—the initiating step in visceral hypersensitivity—is mediated by ion channels such as TRPV1 and TRPA1 expressed on DRG nociceptors.

Substantial evidence indicates that microbial metabolites (e.g., SCFAs, secondary bile acids) and their downstream inflammatory mediators can directly activate or sensitize these ion channels (Zhao et al., 2025). This leads to cation influx (primarily Ca^2+^), causing neuronal depolarization and lowering the activation threshold—thereby inducing peripheral sensitization (Roversi et al., 2021; Zhao et al., 2025). Furthermore, acid-sensing ion channel 3 (ASIC3)—highly sensitive to tissue acidification—may be activated by local pH changes from gut microbiota dysbiosis, further contributing to pain signal generation (Vick and Askwith, 2015; Takeda et al., 2025).

**FIGURE 1:**
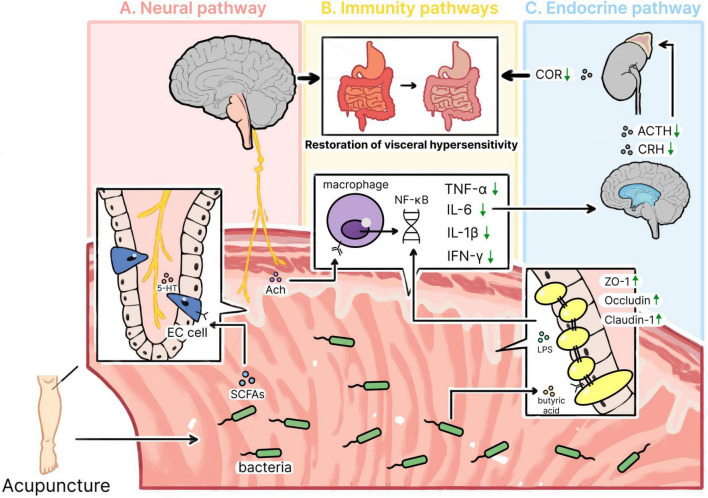
Schematic overview of the multi-pathway mechanisms by which acupuncture modulates the microbiota-gut-brain axis to treat IBS. Acupuncture (e.g., at ST36) remodels gut microbiota composition, increasing production of short-chain fatty acids (SCFAs, including butyrate) and reducing lipopolysaccharide (LPS). These microbial metabolites act through three integrated pathways: **(A)** Neural pathway: SCFAs activate enterochromaffin (EC) cells to release serotonin (5-HT), which stimulates vagal afferents (via 5-HT_3_/5-HT_4_ receptors). Signals ascend to the nucleus tractus solitarius (NTS) and then modulate spinal dorsal horn neurons and brain regions (anterior cingulate cortex, insula), reducing central sensitization and visceral hypersensitivity. Descending vagal efferents release acetylcholine (ACh), which activates the cholinergic anti-inflammatory pathway. **(B)** Immune pathway: Acupuncture upregulates tight junction proteins (ZO-1, Occludin, Claudin-1), restoring intestinal barrier integrity and limiting LPS translocation. It suppresses TLR4/NF-κB signaling and NLRP3 inflammasome activation, reducing pro-inflammatory cytokines (TNF-α, IL-6, IL-1β, IFN-γ). Butyrate promotes Treg differentiation and an anti-inflammatory microenvironment. **(C)** Endocrine pathway: Acupuncture normalizes hypothalamic-pituitary-adrenal **(HPA)** axis hyperactivity by reducing CRH, ACTH, and cortisol. It also restores serotonin synthesis in EC cells by normalizing tryptophan hydroxylase 1 (TPH1) expression. These integrated effects collectively alleviate both gastrointestinal symptoms (abdominal pain, dysmotility) and psychiatric comorbidities (anxiety, depression), exemplifying the “point-to-surface” networked regulatory pattern discussed in Section 6. (Detailed pathway mechanisms are described in Sections 2.1–2.3). ACC, anterior cingulate cortex; ACh, acetylcholine; ACTH, adrenocorticotropic hormone; CRH, corticotropin-releasing hormone; EC, enterochromaffin; HPA, hypothalamic-pituitary-adrenal; LPS, lipopolysaccharide; NF-κB, nuclear factor kappa-B; NLRP3, NLR family pyrin domain containing 3; NTS, nucleus tractus solitarius; SCFA, short-chain fatty acid; TLR4, toll-like receptor 4; TPH1, tryptophan hydroxylase 1; TNF-α, tumor necrosis factor alpha.

These peripherally sensitized DRG neurons continuously transmit enhanced signals to the spinal dorsal horn, where persistent input can trigger central sensitization. This process involves NMDA receptor activation, enhanced intracellular calcium signaling, and interactions with glial cells (Siegel et al., 2006). Consequently, central sensitization alters spinal neuron responsiveness, causing them to overreact to normal or non-noxious intestinal signals (Figure 1A) —a neuroplastic change widely recognized as the core central mechanism of visceral hypersensitivity in IBS.

### 2.2 Endocrine pathway mechanisms: HPA axis dysregulation and modulation by microbial metabolites

Beyond the neural pathway alterations discussed above, endocrine dysregulation represents another key pathological mechanism in IBS. Multiple studies have confirmed that IBS patients exhibit HPA axis hyperfunction or rhythm disruption. Serum cortisol levels are significantly elevated in IBS patients compared to healthy controls, and this elevation correlates positively with symptom severity and visceral hypersensitivity (El-Salhy et al., 2021; Chang et al., 2022). This endocrine abnormality manifests as chronic low-grade activation—a “tonically active” HPA axis—resulting in prolonged non-specific hypervigilance that may persist even without clear external threats (Kennedy et al., 2014b).

The gut microbiota and its metabolites are considered key regulators of HPA axis function. The molecular mechanisms involve synergistic central and peripheral disturbances. Overactivation of hypothalamic corticotropin-releasing hormone (CRH) neurons is a critical initiating step: under chronic stress, excessive CRH stimulates pituitary ACTH secretion, which in turn activates adrenal glucocorticoid release (Brugnatelli et al., 2020; Chen et al., 2022; Aggeletopoulou and Triantos, 2024). Furthermore, elevated serum IL-6 in IBS patients—closely associated with HPA axis hyperactivity—can act on hypothalamic CRH neurons, forming a positive feedback loop (Figure 1B) that amplifies CRH activation (Bethin et al., 2000; Kano et al., 2017; Weng et al., 2024). Gut microbiota dysbiosis is a key driver of this pro-inflammatory cytokine production, linking microbial alterations to endocrine dysregulation.

IBS patients exhibit a prolonged HPA axis response to acute psychological stress. The Trier Social Stress Test (TSST) has shown that, compared to healthy controls, IBS patients have higher peak ACTH and cortisol levels and a slower return to baseline, suggesting impaired negative feedback (Kennedy et al., 2014a; Yuan et al., 2017). Additionally, magnetic resonance spectroscopy has revealed altered limbic-prefrontal connectivity and a reduced glutamate/glutamine ratio, linking central excitatory neurotransmitter imbalance to HPA dysregulation (Weaver et al., 2016).

These HPA axis disturbances also impact local brain-gut peptide networks. Approximately 90% of the body’s serotonin is produced in the gut. In IBS, the number of serotonin-producing enterochromaffin (EC) cells is increased, while serotonin reuptake transporter (SERT) expression is decreased. Together, these changes lead to excessive local serotonin accumulation, contributing to intestinal hypermotility and visceral hyperalgesia (Ikeda et al., 2022; Horn et al., 2025).

### 2.3 Immune pathway mechanisms: from mucosal inflammation to neuro-immune interaction

The understanding of IBS pathophysiology is evolving from a purely “functional” disorder toward a condition with a defined biological basis, with growing recognition that bidirectional gut-brain signaling contributes to its development (Aggeletopoulou and Triantos, 2024). Accumulating evidence further indicates that aberrant immune activation serves as a critical bridge linking gut microbial alterations to both gastrointestinal and central nervous system symptoms (Weaver et al., 2016; Ikeda et al., 2022; Horn et al., 2025).

In this process, gut microbiota dysbiosis is recognized as a key initiating factor that triggers abnormal immune responses. IBS patients exhibit reduced gut microbial diversity, characterized by increased abundance of certain genera (e.g., gas-producing bacteria) and decreased beneficial bacteria, such as SCFA-producing species (Krynicka et al., 2025; Palaniswamy, 2025). This dysbiosis disrupts microecological homeostasis and directly impairs intestinal barrier function (Song et al., 2025; Zhuang et al., 2024). The compromised intestinal barrier becomes more permeable, facilitating translocation of bacterial components (e.g., lipopolysaccharide [LPS]) into the submucosa and persistently activating local immune responses—not as overt acute inflammation, but as a state of low-grade inflammation (Bischoff et al., 2014). Key pathological features include increased mucosal infiltration of immune cells, such as mast cells, eosinophils, and lymphocytes (Song et al., 2025; Zhou L. et al., 2025).

Low-grade inflammation is not an endpoint, but rather the starting point for more complex interactions between immune cells and intestinal sensory neurons. Within this network, mast cells play a pivotal role. Studies confirm that mast cell numbers are increased in the colonic mucosa of IBS patients and are anatomically closer to nerve endings (Barbara et al., 2004; Dothel et al., 2023). Activated mast cells degranulate, releasing mediators including histamine, proteases, serotonin, prostaglandins, and cytokines (e.g., TNF-α, IL-6). These mediators act on corresponding receptors on sensory neurons (e.g., histamine H1 receptors, protease-activated receptors [PARs]), leading to neuronal depolarization and hyperexcitability (Barbara et al., 2007; Buckley et al., 2014; Botschuijver et al., 2019). The persistence of this “neuro-immune interaction” is widely recognized as a key peripheral mechanism (Figure 1C) underlying visceral hypersensitivity in IBS.

## 3 Modulatory effects of acupuncture on the gut microbiota in IBS

Acupuncture has demonstrated efficacy in improving clinical symptoms of IBS. Accumulating evidence suggests that acupuncture’s effects may involve modulation of the dysregulated gut microecosystem. Although studies vary in acupoint protocols and treatment courses, most consistently report that acupuncture corrects gut microbiota dysbiosis in IBS, shifting the microbial structure toward a profile resembling that of healthy individuals.

### 3.1 Changes in community structure and diversity

Alpha and beta diversity of the gut microbiota are core indicators of microecological homeostasis. Although IBS patients often exhibit altered gut microbiota diversity compared to healthy individuals, the direction of change—whether increased or decreased—remains inconsistent across studies and disease subtypes (Altomare et al., 2021; Carcy et al., 2024). Acupuncture research offers a valuable lens for understanding this complexity.

Findings from clinical trials on the effects of acupuncture on gut microbiota alpha diversity in IBS patients have been largely negative. For example, a study in constipation-predominant IBS (IBS-C) found that among treatment responders, alpha diversity did not change significantly during treatment (Yaklai et al., 2025). This lack of change may be context-dependent, suggesting that acupuncture’s clinical efficacy may not rely on a general increase in alpha diversity, but rather on selective remodeling of overall microbial structure (beta diversity) and specific functional populations. Notably, when baseline alpha diversity is highly heterogeneous, this indicator may be insufficiently sensitive to treatment response.

At the preclinical level, acupuncture has been shown to significantly increase alpha diversity in stress-induced IBS mouse models (Mengzhu et al., 2022). Additionally, studies in Crohn’s disease patients have shown that acupuncture improves their reduced alpha diversity (Bao et al., 2022). These findings across models and populations suggest that acupuncture’s effect on alpha diversity is not a universal increase, but rather a restoration of compromised baseline states. Future studies with larger sample sizes and subgroup analyses based on baseline characteristics are warranted to clarify this issue (Qi et al., 2022a).

In contrast to alpha diversity, the effects of acupuncture on gut microbiota beta diversity are more consistent and pronounced. Studies consistently show that the overall gut microbiota structure in IBS patients differs significantly from that of healthy individuals, and acupuncture reduces this difference—i.e., decreases the beta diversity distance between IBS patients and healthy controls (Rodriguez Paris et al., 2022; Yaklai et al., 2025). A key study on electroacupuncture in constipation-predominant IBS (IBS-C) further revealed that although patients’ microbial structure remained similar to baseline at treatment completion, after 1 month of follow-up, the overall microbial composition had significantly converged toward that of healthy controls (Yaklai et al., 2025). This finding has important implications: acupuncture’s initial therapeutic effects may not depend on immediate microbial restructuring; rather, the post-treatment convergence toward a healthy profile may represent the microecological basis for consolidation and maintenance of efficacy, reflecting the long-term ecological impact of treatment. This capacity to remodel the overall microbial ecological landscape is considered a core mechanism by which acupuncture restores gut microecological balance and produces sustained therapeutic effects.

### 3.2 Modulation of beneficial bacterial genera

Acupuncture not only remodels overall gut microbiota structure but also targets specific beneficial bacteria. Butyrate-producing genera are primary targets of acupuncture’s modulatory effects. *Faecalibacterium prausnitzii* (*F. prausnitzii*), a key butyrate-producing bacterium with anti-inflammatory properties, is frequently decreased in IBS patients (Chen et al., 2023; Di Pierro et al., 2025). Acupuncture has been shown to effectively increase its abundance. For example, electroacupuncture at Zusanli (ST36) increases the relative abundance of butyrate-producing bacteria, including *F. prausnitzii* (Liu Y. L. et al., 2023; Jiang et al., 2024). Studies in inflammatory bowel disease (IBD) similarly report that acupuncture improves *F. prausnitzii* abundance, outperforming placebo controls (Bao et al., 2019). This increase is critical for suppressing intestinal inflammation and promoting barrier repair.

Similarly, *Bifidobacterium*—a core probiotic genus—is often reduced in IBS patients, and acupuncture effectively ameliorates this reduction (Bao et al., 2019, 2022; Chen et al., 2023; Di Pierro et al., 2025). This shift helps regulate intestinal pH, inhibit potential pathogens, and improve host immune function.

Acupuncture also inhibits harmful bacteria. Escherichia-Shigella—a genus encompassing multiple potential pathogens—is closely associated with intestinal inflammation and barrier disruption when overgrown. Currently, direct clinical evidence for acupuncture-induced reduction of this genus in IBS patients remains limited. However, evidence from animal models, case series, and some clinical trials collectively suggests an inhibitory effect of acupuncture on these bacteria. For example, animal studies show that *F. prausnitzii* supplementation—which acupuncture promotes—significantly reduces Escherichia-Shigella abundance (Qin et al., 2025). Additionally, several studies report decreased Enterobacteriaceae abundance following acupuncture or electroacupuncture, and this change parallels improvement in IBS symptoms.

### 3.3 Functional predictions of metabolic pathways: focus on SCFA synthesis

The functional characteristics of the gut microbiota are often considered to provide deeper insight into host-microbe interactions than taxonomic composition alone. Emerging evidence suggests that acupuncture modulates host metabolism by regulating the gut microbiota.

Acupuncture may enhance SCFA biosynthesis. SCFAs—particularly butyrate—are critical for maintaining intestinal epithelial health, suppressing inflammation, and regulating gut motility, and their levels are often dysregulated in IBS patients. Functional prediction analyses confirm that acupuncture upregulates microbial pathways associated with SCFA synthesis (Yan et al., 2023; Yaklai et al., 2025). This finding aligns with the previously discussed increase in butyrate-producing bacteria (e.g., *F. prausnitzii*) (Chen et al., 2023; Liu Y. L. et al., 2023; Di Pierro et al., 2025), supporting the notion that acupuncture restores intestinal SCFA production by promoting key functional populations, thereby improving the local gut environment.

Furthermore, acupuncture may reshape microbiota-mediated tryptophan metabolism. Tryptophan is a common precursor for serotonin and kynurenine synthesis. The balance of its metabolic flux directly influences intestinal sensation, motility, and even emotional states—a pathway frequently disrupted in IBS patients. A study using PICRUSt2 analysis in constipation-predominant IBS (IBS-C) identified significant abnormalities in gut microbial tryptophan metabolism before treatment. After electroacupuncture, the functional abundance of pathways—including tryptophan biosynthesis, the kynurenine pathway, and histidine degradation—shifted toward normalization, converging to levels resembling those of healthy controls (Yaklai et al., 2025). This suggests that acupuncture may regulate intestinal 5-HT levels by remodeling microbial metabolic function. Such modulation likely helps ameliorate brain-gut axis dysregulation, offering a microbiota-mediated mechanism for acupuncture’s dual efficacy in alleviating both physical and psychological symptoms in IBS.

At a macro level, the clinical response to acupuncture in IBS correlates significantly with the degree of gut microbiota structural optimization: the closer the post-intervention microbial structure approximates a healthy state, the more pronounced the symptom improvement (Jiang et al., 2024). This association supports the notion that acupuncture’s therapeutic effects arise not from a single target, but from systemic modulation of the gut microecological network, underpinning its comprehensive benefits in IBS management.

## 4 Animal experimental evidence for acupuncture in regulating the microbiota-gut-brain axis to treat IBS

Under controlled conditions, animal studies have consistently confirmed that acupuncture regulates the gut microbiota. Furthermore, they have revealed downstream restorative effects, elucidating potential causal mechanisms.

### 4.1 Acupuncture ameliorates behavioral and visceral hypersensitivity in IBS

IBS is frequently comorbid with anxiety and depression, and its core feature of visceral hypersensitivity (VHS)—a reduced pain threshold to colorectal distension—is well validated in animal models. Notably, improvements in behavior and visceral sensation often correlate. Acupuncture, a neuromodulatory intervention, effectively alleviates anxiety-like behavior in IBS rats. In the open field test (OFT) and elevated plus-maze (EPM), IBS models exhibit pronounced anxiety: increased time in the peripheral zone and reduced time in the central area (OFT), along with decreased open-arm entries and time (EPM) (Yu et al., 2019; Hou et al., 2022; Zhan et al., 2022). EA at ST36 reverses these indicators, increasing exploratory activity and reducing anxiety.

Acupuncture significantly reduces VHS in IBS rats, assessed by two complementary techniques. The abdominal withdrawal reflex (AWR) score evaluates visceral sensitivity by scoring (0–4) abdominal contractions in response to colorectal distension; higher scores indicate greater VHS (Zhu L. et al., 2015; Zhou X. et al., 2022). Electromyography (EMG) provides an objective, quantitative measure by recording abdominal muscle activity during distension and calculating the area under the curve (Jia et al., 2013; Zhang L. et al., 2018; Altomare et al., 2021).

In the AWR assessment, acupuncture-treated rats had significantly lower scores at the same distension pressure than model rats, indicating an increased pain threshold (Tian et al., 2008; Zhou et al., 2012; Altomare et al., 2021). Similarly, EMG revealed that EA significantly reduced the area under the curve of distension-evoked abdominal muscle activity. These physiological improvements provide experimental evidence for acupuncture’s analgesic effects in IBS (Chen et al., 2021d; Rodríguez-Sojo et al., 2022; Qu et al., 2024). The underlying mechanisms may involve multilevel modulation of peripheral and central nervous systems, including regulation of spinal neurotransmitter release and inhibition of overactivation in brain regions such as the anterior cingulate cortex (Baseer et al., 2014).

More importantly, the improvements in anxiety-like behavior and visceral hypersensitivity were statistically correlated. As a classic brain-gut interaction disorder, IBS features a bidirectional relationship between anxiety and visceral hypersensitivity, potentially forming a reciprocal causal loop. In a maternal separation-induced IBS rat model, a significant negative correlation was found between the percentage of time spent in the OFT central zone and AWR scores (Wu et al., 2020). This suggests that rats with more pronounced anxiety-like behavior (less central zone time) tend to have greater visceral hypersensitivity (higher AWR scores) (Jansson-Knodell et al., 2022). For non-normally distributed or ordinal data, Spearman’s rank correlation analysis is appropriate for assessing the strength and significance of associations (Bai et al., 2023).

Although studies have reported acupuncture’s benefits on anxiety and VHS separately, few have explicitly analyzed their correlation. Notably, after treatment, anxiety and VHS maintained a negative correlation, but the overall data distribution improved. Moreover, their improvement magnitudes were significantly positively correlated, indicating synchronized improvement.

Acupuncture modulates the ascending pathways that transmit information from the gut to the brain. On one hand, it suppresses excessive visceral nociceptive signal transmission; on the other, it regulates the limbic system’s emotional processing of these signals, thereby alleviating both visceral hypersensitivity and anxiety. Another major ascending pathway for visceral nociceptive signals is the spinothalamic tract. According to the gate control theory and the theory of somatic-visceral convergence, non-noxious somatic sensory signals (primarily conducted via Aβ and Aδ fibers) from acupuncture points such as ST36 (Zusanli) also enter the spinal dorsal horn, where they interact with visceral nociceptive signals (Fan et al., 2020; Wang Y. L. et al., 2023; Yang et al., 2023). By activating inhibitory interneurons or presynaptic inhibition, acupuncture signals can “close” or attenuate the transmission of visceral nociceptive signals to the brain, thereby achieving analgesic effects at the spinal level and directly relieving visceral hypersensitivity.

Once modulated by acupuncture, the brain sends regulatory signals back to the gut via descending pathways, repairing the intestinal barrier and activating the cholinergic anti-inflammatory pathway. This effectively inhibits the production and release of pro-inflammatory cytokines, thereby reducing neuroinflammation and interrupting the vicious cycle. Consequently, intestinal function and the microenvironment are further improved, establishing avirtuous cycle.

This synchronized improvement suggests that acupuncture’s effects in IBS models are not symptom-specific but reflect holistic regulation of brain-gut dysfunction. Acupuncture alters the brain’s perception and emotional processing of visceral signals by activating ascending pathways (e.g., the vagus nerve) and modulating spinal signal processing. Simultaneously, by reshaping the functional connectivity of higher brain centers, it sends descending regulatory commands via the vagal efferent pathway and the HPA axis, thereby improving local intestinal inflammation, barrier function, and microbial ecology. These findings provide behavioral evidence for acupuncture’s integrated efficacy via the MGB axis.

### 4.2 Restoration and enhancement of intestinal barrier function by acupuncture

While the restoration of the physical barrier is a downstream effect of electroacupuncture, its regulation of the MGB immune pathway acts as the upstream key mechanism. In IBS, expression of key tight junction proteins is often decreased, leading to structural loosening and impaired barrier function (D’Antongiovanni et al., 2020). Substantial evidence from animal models indicates that EA can reverse this process. For example, in diarrhea-predominant IBS models, EA promotes SCFA-producing bacteria, providing butyrate and other metabolites that fuel intestinal epithelial cells. This is accompanied by upregulated expression (protein and/or mRNA) of ZO-1, Occludin, and Claudin-1 in colonic tissue (Wei et al., 2019; Mengzhu et al., 2023), reducing intestinal permeability and decreasing endotoxin (e.g., LPS) translocation (Hao et al., 2022; Hou et al., 2025). These molecular changes provide direct evidence for EA-mediated restoration of intestinal barrier function. Morphological studies support this conclusion: immunofluorescence has demonstrated that EA-treated IBS rats exhibit improved continuity and integrity of tight junction structures between intestinal epithelial cells (Liu S. et al., 2023; Liu S. et al., 2025).

The intestinal barrier, functioning like a “zipper,” tightly regulates paracellular permeability (Liao et al., 2008; Soliman et al., 2018; Ma M. et al., 2025). In IBS animal models, intestinal permeability is often assessed by serum D-lactate and diamine oxidase (DAO). D-lactate is a bacterial metabolite, while DAO is localized in intestinal epithelial cells; elevated levels of both indicate barrier disruption. Increased permeability facilitates entry of bacterial components (e.g., LPS) into the circulation, potentially triggering systemic low-grade inflammation (Liao et al., 2008; Soliman et al., 2018). By restoring barrier integrity and reducing permeability, EA decreases this pro-inflammatory translocation at its source. Studies show that EA significantly reduces serum D-lactate in IBS models, providing direct evidence for reduced intestinal permeability (Zhu M. F. et al., 2015; Hao et al., 2022).

Furthermore, EA-induced upregulation of tight junction proteins involves complex intracellular signaling networks, not isolated molecular events. Toll-like receptor 4 (TLR4), a key LPS receptor, activates downstream NF-κB signaling and the NLRP3 inflammasome, leading to release of pro-inflammatory cytokines (e.g., TNF-α, IL-1β, IL-6) (Cao et al., 2025; Wang D. et al., 2025). These cytokines can further disrupt tight junctions, potentially creating a self-reinforcing cycle of “inflammation—barrier disruption—more inflammation” (Liu S. et al., 2023). Current evidence shows that acupuncture targets multiple nodes in this cascade. Specifically, it downregulates TLR4, MyD88, and NF-κB in IBS models (Qiao et al., 2020; Zhai et al., 2020; Chen et al., 2021a; Wang D. et al., 2023), while concurrently inhibiting NLRP3 inflammasome activation and reducing IL-1β/IL-18 release (Huang et al., 2022; Zhou F. et al., 2022). Although direct comparative studies are lacking to determine the most critical target (Wan et al., 2024; Su et al., 2025), it is clear that EA’s comprehensive suppression of this inflammatory pathway helps interrupt the vicious cycle (Liu Z. et al., 2022; Huang et al., 2023; Li et al., 2024).

This barrier restoration, coupled with an improved inflammatory microenvironment, synergistically reduces bacterial translocation and dissemination of microbial metabolites to peripheral organs (e.g., mesenteric lymph nodes, liver). A study using a sepsis-induced intestinal injury model found that EA reduced bacterial translocation, providing indirect evidence for similar protective effects in IBS (Zhu M. F. et al., 2015; Wu et al., 2017; Zhang Z. et al., 2018). Acupuncture reduces intestinal permeability by directly repairing the physical barrier. More importantly, its anti-inflammatory effects—via inhibition of TLR4/NF-κB/NLRP3 pathways—help break the persistent low-grade inflammatory cycle in IBS, creating a favorable microenvironment for long-term maintenance of barrier function.

### 4.3 Remodeling effects of acupuncture on gut microbiota structure

Gut microbiota dysbiosis is a common pathological feature across various animal models of irritable bowel syndrome (IBS). Accumulating evidence indicates that acupuncture’s therapeutic effects extend beyond neural regulation to significantly remodel the gut microbiota’s composition and function, establishing a functional bridge between neuromodulation and microecological restoration. The MGB axis thus serves as an integrative framework for understanding the complex mechanisms of acupuncture in IBS.

Multiple IBS animal models consistently exhibit a significant decrease in gut microbiota alpha diversity (Qin et al., 2010; Mengzhu et al., 2022; Zhang et al., 2023). Numerous studies confirm that acupuncture or electroacupuncture (EA) can reverse this trend. For example, EA at Zusanli (ST36) significantly increases microbial diversity and richness in IBS rat models, restoring them to levels approaching those of healthy controls (Wang L. et al., 2020; Hu et al., 2021; Mengzhu et al., 2022). This suggests that acupuncture can restore impaired microbial ecological stability in IBS. However, not all studies report significant alpha diversity changes. For instance, a clinical study in IBS-C patients found that despite symptom improvement, gut microbiota alpha diversity (Shannon index) did not change significantly after treatment (Yaklai et al., 2025).

In contrast to alpha diversity, changes in beta diversity offer a more macroscopic view of how acupuncture remodels overall gut microbiota structure. Visualization results based on principal coordinate analysis (PCoA) have shown that the microbial structure of IBS model animals typically separates from that of healthy controls, forming distinct clusters. A series of studies have consistently observed that acupuncture or electroacupuncture treatment significantly alters the beta diversity of the gut microbiota in IBS model animals, promoting a shift in overall structure toward that of healthy controls (Mengzhu et al., 2022; Houshyar et al., 2023). For example, in a DSS-induced colitis mouse model, PCoA showed that the electroacupuncture group had a microbial profile closest to that of healthy controls among all experimental groups (Liu G. H. et al., 2022).

At the taxonomic level, acupuncture corrects key bacterial imbalances, modulating abundance at both the phylum and genus levels. At the phylum level, the gut microbiota consists mainly of Firmicutes and Bacteroidetes; their ratio (F/B ratio) is a macroscopic indicator of gut health. This ratio is often elevated in metabolic diseases and IBS animal models (Barandouzi et al., 2021; Lu et al., 2024). Multiple studies consistently show that acupuncture reduces the abnormally elevated F/B ratio in IBS models (Yaklai et al., 2025), typically by increasing Bacteroidetes abundance, decreasing Firmicutes, or both (Wang et al., 2019). Thus, normalization of the F/B ratio represents key evidence for acupuncture-induced macrostructural remodeling of the gut microbiota.

For instance, in the maternal separation model, rats exhibit increased Bacteroidetes and decreased Firmicutes (Wang et al., 2018; Meza Ocampos et al., 2023). EA at ST36 reverses this dysregulation, normalizing the F/B ratio and significantly increasing probiotic *Lactobacillus* (Mengzhu et al., 2022). In chemically induced models (e.g., acetic acid enema), detailed quantitative data remain limited, but the mode of action is likely similar. Existing studies suggest that EA increases butyrate-producing bacteria (e.g., Butyricimonas) while inhibiting potential pathogens such as Campylobacter and Helicobacter (Wang et al., 2019; Barandouzi et al., 2021; Lu et al., 2024), helping restore intestinal metabolic function and suppress inflammation.

Additionally, increased Proteobacteria abundance is a recognized marker of dysbiosis and inflammation (Lupp et al., 2007; Shin et al., 2015). Observed in some IBS models, this elevation is significantly reduced by acupuncture (Lv et al., 2022; Cheng et al., 2024; Qi et al., 2025; Yan et al., 2025), mitigating pathogenic and inflammatory risks.

At the genus level, acupuncture modulates the gut microbiota with greater precision, exhibiting a bidirectional regulatory pattern. On one hand, microbial metabolites directly modulate neural signaling. Acupuncture increases SCFA-producing bacteria (e.g., *Lactobacillus* and *Bifidobacterium*), which ferment dietary fiber to produce butyrate, propionate, and other SCFAs (Yan et al., 2025). Notably, acupuncture significantly increases *Lactobacillus* abundance (Zhang et al., 2021; Ma K. et al., 2025), a key probiotic that produces lactic acid, inhibits pathogens, and modulates host immunity. SCFAs act as signaling molecules, activating G-protein-coupled receptors (GPR41/43) on vagal afferent terminals, converting local chemical signals into neural impulses. These signals, transmitted to the nucleus tractus solitarius, modulate gastrointestinal motility and sensation via descending autonomic pathways (Chen et al., 2025; Wu et al., 2025). Studies suggest that EA at ST36 activates the vagus nerve in part through SCFAs derived from microbiota modulation (Ma K. et al., 2025). Thus, acupuncture’s role in increasing these bacteria is critical for restoring gut microecological balance.

These findings suggest that acupuncture does not simply inhibit or promote all bacteria indiscriminately. Instead, it selectively promotes beneficial bacteria while suppressing the overgrowth of harmful bacteria (Yan et al., 2023), thereby achieving precise remodeling of the gut microbiota structure. This microbiota-mediated mechanism provides a neurobiological basis for acupuncture’s dual efficacy in alleviating both abdominal pain and bowel habit abnormalities in IBS patients.

### 4.4 Establishing the causal role of microbiota in acupuncture’s effects

Although numerous studies have revealed associations between acupuncture, microbiota changes, and symptom improvement, establishing causality is essential to determine whether the gut microbiota mediates acupuncture’s therapeutic effects. Logically, if acupuncture’s effects are driven by microbiota changes, then transplanting “healthier” microbiota from treated donors to untreated recipients should partially reproduce the therapeutic benefits (Xing et al., 2025a). Fecal microbiota transplantation (FMT) provides a powerful tool for testing this causality (Xie et al., 2025).

Studies in autism spectrum disorder (ASD) models exemplify this approach: EA ameliorates social deficits and anxiety-like behaviors in ASD mice, accompanied by significant gut microbiota alterations (Strati et al., 2017; Chen et al., 2025). To test causality, researchers transplanted fecal microbiota from EA-treated ASD donors (with symptom improvement) into untreated ASD recipients. Recipient mice also showed improved social behavior and reduced neuroinflammation (Strati et al., 2017; Ye et al., 2025), indicating that EA-induced microbiota changes possess therapeutic potential independent of the acupuncture procedure (Wang J. et al., 2023; Feng et al., 2025).

A reverse experiment—transplanting microbiota from untreated ASD donors into EA-treated recipients—significantly attenuated EA’s therapeutic effects (Chen et al., 2025), confirming that the gut microbiota is an indispensable mediator of acupuncture’s efficacy; disrupting it reduces treatment effects.

Establishing causality requires not only demonstrating that microbiota transfer confers benefits, but also proving necessity—that is, in the absence or depletion of gut microbiota, acupuncture’s therapeutic effects should be diminished or abolished. This hypothesis has been tested using antibiotic-treated and germ-free animal models (Curtis et al., 2014; Jiang et al., 2023). In these models, microbiota depletion provides a controlled system to assess whether an intact microbial community is required for acupuncture to exert its effects (Wang S. et al., 2020; Tong et al., 2022; Deng et al., 2023; Gargiulo et al., 2025).

However, the application of fecal microbiota transplantation (FMT) in acupuncture research remains in its preliminary stages, and several methodological challenges limit current interpretations (Robles Vera, 2020; Tang et al., 2025b). Donor selection criteria, fecal sample processing methods, and transplantation parameters such as dose, frequency, and administration route vary considerably across studies, and standardized protocols are lacking (Arnoriaga-Rodríguez et al., 2020; Arnoriaga-Rodríguez et al., 2021; Baruch et al., 2021; Bokoliya et al., 2021; Hui et al., 2022; Mayneris-Perxachs et al., 2022; Sharma et al., 2022; Mingaila et al., 2023; Yang et al., 2026). These variations hinder direct comparison of results and limit the reproducibility of findings. Furthermore, host factors including animal strain, age, sex, housing conditions, and diet critically influence gut microbiota composition and must be rigorously controlled to ensure that observed effects are attributable to acupuncture-induced microbial changes rather than confounding variables (Secombe et al., 2021; Prado et al., 2022; Mingaila et al., 2023; Huang et al., 2025). Addressing these methodological issues will be essential for advancing causal evidence in future studies.

### 4.5 Limitations and translational challenges of iBS-related animal experiments

Animal experiments serve as a critical bridge connecting clinical observations with mechanistic exploration, playing an essential role in validating acupuncture’s therapeutic efficacy and elucidating its biological mechanisms. Given that gut microbiota dysbiosis is considered a core pathogenic factor in IBS, an ideal animal model of IBS should adequately recapitulate the characteristics of microbial dysbiosis observed in human patients. However, current evidence indicates that different modeling methods induce distinct patterns of gut microbiota alterations, which directly contributes to substantial heterogeneity in the results of acupuncture intervention studies based on different models.

For example, a comparative study analyzing two stress-induced IBS rat models—maternal separation and multiple early-life adversity—found that although both induced significant gut microbiota alterations, the specific patterns of change and the metabolic pathways involved differed markedly (Enqi et al., 2021). This finding suggests that even among models classified under the “stress” type, the underlying pathological basis of microbiota dysbiosis may vary. Furthermore, the duration of the modeling procedure itself is a critical factor influencing microbiota structure. Similar patterns have been observed in IBS research; for instance, in neonatal maternal separation (NMS)-induced IBS, the length of the separation period significantly affects the specific pattern of gut microbiota dysbiosis. Rats subjected to NMS on postnatal days 2–14 versus days 2–21 exhibit significant differences in alpha diversity, the Bacteroidetes/Firmicutes ratio, and the abundance of specific genera, with longer separation periods resulting in more stable dysbiosis patterns that persist into adulthood (Tao et al., 2023). This aligns with findings in other disease models, such as polycystic ovary syndrome (Zhao et al., 2021). Therefore, the inherent heterogeneity of animal models and the consequent variability in gut microbiota profiles constitute a major challenge for effectively integrating and comparing results across acupuncture studies in IBS.

Among the various modeling methods, acetic acid enema is widely used due to its relative simplicity and high success rate in model establishment. However, as a potent chemical stimulus, the pathological changes induced by this method inherently differ from the natural course of chronic IBS in humans. Acetic acid is corrosive; low-concentration enemas can directly damage the colonic mucosal epithelium, triggering acute focal inflammation and subsequently inducing significant perturbations in the gut microbiota. Studies have shown that in a diarrhea-predominant IBS rat model induced by acetic acid enema combined with restraint stress, the fecal microbiota structure exhibits decreased abundance of beneficial bacteria such as *Lactobacillus* and *Bifidobacterium*, accompanied by increased abundance of Blautia (Jia et al., 2013; Zhu L. et al., 2015; Zhou X. et al., 2022). This pattern of microbiota alteration, directly driven by acute chemical injury, more closely resembles the microbial response observed after acute enteritis and may differ substantially from the chronic, low-grade, and diverse dysbiosis characteristic of human IBS. Therefore, when using the acetic acid enema model to evaluate acupuncture efficacy, the “high-intensity insult” inherent to the modeling procedure itself becomes a significant confounding factor, potentially interfering with accurate assessment of acupuncture’s “true” therapeutic effects.

Acupuncture and moxibustion have been demonstrated to possess anti-inflammatory and mucosal protective properties. Thus, the therapeutic effects observed in the acetic acid enema model—such as amelioration of diarrhea and reduction of visceral hypersensitivity—may partially stem from acupuncture’s inhibition of acute inflammation induced by acetic acid and its reparative effects on mucosal damage. Indeed, studies have found that in this model, acupuncture intervention promotes the proliferation of probiotics such as *Bifidobacterium* and Akkermansia, while inhibiting potential pathogens like Helicobacter and Prevotella. However, these observed therapeutic effects may partly reflect acupuncture’s general reparative effects on acute chemical injury rather than its specific regulatory role in IBS pathophysiology. This raises a risk of false positive interpretations when using strong-stimulus models like acetic acid enema. Such models offer clear pathological phenotypes but have limited external validity. Thus, conclusions about acupuncture’s modulation of gut microbiota in IBS based on these models require cautious interpretation.

While model heterogeneity is an inherent limitation of animal studies, the lack of standardization in acupuncture parameters is an acquired bottleneck that hinders scientific validation. Acupuncture is a multi-parameter composite intervention, with key variables including acupoint selection, stimulation intensity, frequency, waveform, and treatment duration. Existing research indicates that variations in these parameters can influence therapeutic outcomes (Tang et al., 2025a). Current animal experiments often lack unified standards and systematic optimization in the selection of these parameters, which not only reduces the reproducibility of research findings but also hinders the establishment of clear dose-effect relationships.

Acupuncture exerts its synergistic therapeutic effects as a potent “microecological modulator” and “neuroimmune regulator.” Its therapeutic action is not linear or singular but rather a multi-target, multi-pathway, dynamically interactive network effect. By restoring the health of the gut microbiota—an “endogenous organ”—acupuncture triggers the homeostatic recovery of the entire microbiota-gut-brain (MGB) axis and the neuroimmune network. However, because it is often difficult to accurately distinguish whether the observed effects represent a general action of acupuncture or incidental phenomena arising from specific parameter combinations, advancing the research paradigm in animal studies from correlational description to causal validation is considered an important approach to improving the scientific rigor of acupuncture mechanism research and facilitating its potential clinical translation.

## 5 Clinical trial evidence for acupuncture in regulating gut microbiota to treat IBS

### 5.1 Macro-level modulation: alpha and beta diversity

Clinical studies have validated acupuncture’s capacity to modulate gut microecological structure, assessed by two core indicators: alpha diversity and beta diversity. Early animal and other-disease studies suggested that acupuncture can increase alpha diversity, but this effect has not been consistently observed in IBS-specific trials. For example, a study in IBS-C patients found that among electroacupuncture “responders” (with significant symptom improvement), alpha diversity (Shannon index) showed no significant change from baseline (Botschuijver et al., 2019; Yang et al., 2025). This finding suggests that, in this specific clinical context, symptom improvement may not directly depend on a general increase in alpha diversity. Nevertheless, this inconsistency may reflect clinical heterogeneity—variations in baseline microbiota, disease subtypes, protocols, and sample sizes—that could obscure effects in specific subgroups.

Another IBS-C study reported no significant differences in alpha diversity (Shannon, Chao1, observed species) pre-to-post treatment or versus healthy controls (Vervier et al., 2022; Bora et al., 2023; Liu et al., 2024), challenging the notions that IBS universally reduces alpha diversity and that acupuncture works primarily by restoring it. Indeed, a meta-analysis confirmed literature inconsistency, with some studies reporting decreases, others no change, and a few increases (Bora et al., 2023). This may indicate that alpha diversity changes are not universal across IBS subtypes or may stem from heterogeneity in design, population, and methods. Current evidence suggests that whether alpha diversity changes may be less critical than understanding when and why differences occur. Acupuncture’s therapeutic mechanisms may be more stably reflected in directional remodeling of overall structure and specific functional populations rather than unidirectional alpha diversity changes.

Multiple clinical studies using beta diversity analysis have consistently shown that, in principal coordinate analysis (PCoA) plots, baseline samples from IBS patients typically form a distinct cluster separated from healthy controls, confirming significant gut microbiota dysregulation (Rajilić-Stojanović et al., 2011; Li M. et al., 2020). After acupuncture, patient samples shift significantly and tend to converge with the healthy control group (Lam et al., 2025). Other studies similarly report “normalization” of microbiota structure after electroacupuncture (Botschuijver et al., 2019), indicating that acupuncture guides a directional shift toward a healthy state. This “convergent remodeling” implies that acupuncture’s effects are not random but represent directional, holistic ecological restructuring. Thus, beta diversity evidence for convergent remodeling is the most compelling clinical evidence supporting acupuncture’s holistic, directional modulation of the gut microbiota in IBS.

### 5.2 Bidirectional modulation of specific bacterial genera

Beyond macro-level shifts, acupuncture’s modulation is more profoundly reflected in precise, bidirectional alterations of specific functional genera. This “bidirectional regulation”—differentially promoting or inhibiting bacteria based on function—underpins its microecological remodeling and therapeutic effects.

Clinical studies consistently show that acupuncture selectively enriches beneficial bacteria essential for gut health. These bacteria—particularly *Lactobacillus* and *Bifidobacterium*, the most frequently reported beneficiaries—play core roles in protecting the intestinal barrier, modulating immunity, and synthesizing nutrients. Multiple studies across different IBS subtypes have observed significant increases in the abundance of these two bacterial genera in patient feces following acupuncture treatment (Xu et al., 2013; Hou et al., 2014; Xu et al., 2017). They inhibit pathogens by producing lactic acid to lower gut pH, and interact with the host immune system to exert anti-inflammatory effects. Notably, transcutaneous auricular vagus nerve stimulation (taVNS)—mechanistically akin to acupuncture—also increases *Lactobacillus* and *Bifidobacterium* in IBS-C patients (Sahn et al., 2023), suggesting that acupuncture may activate vagal pathways to create a favorable environment for these probiotics.

Furthermore, acupuncture’s modulation of SCFA-producing bacteria is especially important, as SCFAs play a central role in maintaining the intestinal barrier, suppressing inflammation, and mediating gut-brain signaling (Ju et al., 2024). Studies show that acupuncture increases overall SCFA-producer abundance in IBS patients. Notably, changes in Senegalimassilia correlate positively with symptom improvement in electroacupuncture-treated IBS-C patients (Elsenbruch and Orr, 2001; Muscatello et al., 2010; Fang et al., 2025), suggesting it may be a key mediator of acupuncture’s effects. This raises the possibility of Senegalimassilia as a predictive biomarker for treatment response (Jarrett et al., 2008), potentially identifying “high responders” via pre-treatment fecal testing.

Acupuncture also suppresses potential pathogens. Multiple studies report decreased abundance of Escherichia coli or the broader Enterobacteriaceae family after acupuncture, helping reduce the antigenic burden driving low-grade inflammation (Song et al., 2019; Zhang et al., 2022). The genus Bacteroides is functionally diverse; not all members are harmful, but some (e.g., *B. fragilis*) may promote inflammation. Other studies report post-acupuncture decreases in potentially harmful bacteria such as Yersiniaceae (Zheng et al., 2021; Liu Y. et al., 2025).

This bidirectional pattern has important implications: acupuncture likely modulates the host’s internal environment—neurotransmitters, hormones, immune state—to selectively favor beneficial bacteria while suppressing harmful niches. From a microbiological perspective, this provides a “niche selection” mechanism for acupuncture’s regulation of the gut microecosystem.

### 5.3 From correlation to mechanism: linking microbiota changes to clinical outcomes

Confirming acupuncture alters gut microbiota is important, but directly linking these changes to symptom improvement has greater clinical value.

Multiple studies report that increases in beneficial bacteria (e.g., *Lactobacillus*, *Bifidobacterium*, *Coprococcus*) correlate negatively with IBS-SSS reductions (symptom alleviation) and positively with IBS-QoL improvements (quality of life) (Botschuijver et al., 2019; Shanshal et al., 2023; Hearn-Yeates et al., 2024). Conversely, decreases in potentially harmful bacteria (e.g., Enterobacteriaceae) also associate with symptom relief (Shanshal et al., 2023).

Further advancing beyond post-treatment correlation analyses, some studies have attempted to explore pre-treatment predictive factors. By constructing complex statistical models, researchers have successfully identified specific baseline bacterial genera that predict acupuncture efficacy. For example, a study on electroacupuncture for constipation-predominant IBS found that the baseline abundance of Senegalimassilia in pre-treatment fecal samples, combined with the magnitude of change in its abundance after treatment, could significantly predict patient clinical response (Botschuijver et al., 2019; Gryaznova et al., 2024; Hill et al., 2024). This implies that not all IBS patients benefit equally; the patient’s own microbiota “background” may be a critical prerequisite for efficacy.

Stratifying patients into “responders” and “non-responders” offers another approach to linking microbiota changes with outcomes. Even with identical treatment, responders and non-responders exhibit distinct post-treatment microbiota patterns (Qi et al., 2022b), indicating that successful efficacy is tied to a specific remodeling trajectory (Wu et al., 2024). These associations suggest that baseline microbiota characteristics or dynamic change patterns may underpin acupuncture’s effects, providing a basis for personalized treatment strategies.

To further elucidate mechanisms, the microbiota’s impact extends beyond composition to its “functional metagenome.” Omics analysis has advanced from describing “who is there” to revealing “what the microbiota does”—remodeling of metabolic functions (Yao et al., 2024; Wang X. et al., 2025). Acupuncture induces significant reconstruction of key metabolic pathways in IBS patients, linking functional changes to MGB axis dysregulation.

How is this beneficial remodeling initiated? An increasingly recognized mechanism is that acupuncture optimizes the microbial environment via neuro-immune pathways. Stimulating specific acupoints (e.g., ST36) activates vagal afferents (Yang et al., 2021; Dong et al., 2025; Xing et al., 2025a); signals transmitted to the nucleus tractus solitarius then modulate gastrointestinal function via efferent vagal pathways. IBS patients exhibit gut low-grade inflammation, an unfavorable microenvironment for beneficial bacteria. By activating the vagus nerve and the cholinergic anti-inflammatory pathway, acupuncture suppresses local inflammation, creating a favorable ecological niche that promotes beneficial bacteria while suppressing harmful ones—achieving “beneficial” microbiota remodeling.

### 5.4 Clinical implications and patient selection

Patients with refractory or intolerant IBS are the most core and clearly defined candidates for acupuncture, as they have, for various reasons, failed to find satisfactory solutions within conventional medical systems. Many IBS patients experience limited symptom improvement after trying dietary modifications (e.g., low FODMAP diet), antispasmodics, antidiarrheals, prokinetics, or neuromodulators. For these patients defined as having “refractory IBS,” acupuncture offers a novel approach with a distinct mechanism of action. A study on patients with refractory IBS reported that adding acupuncture to conventional treatment significantly improved the Symptom Severity Score (IBS-SSS), with 83% of patients experiencing a reduction to mild symptom severity. Although the same study observed no between-group statistical differences in IBS-QOL or depression scores (PHQ-9), the marked improvement in overall symptoms is itself an important component of enhanced quality of life (Yang et al., 2025). Conversely, other high-quality trials have demonstrated that acupuncture significantly improves IBS-specific quality of life (IBS-QOL), with verum acupuncture showing greater improvement than control interventions. Because acupuncture acts by holistically modulating the brain-gut axis rather than targeting a single symptom, it may bring breakthroughs for these patients who are otherwise stuck. For instance, one study demonstrated that patients in the verum acupuncture group showed a significant increase in IBS-specific quality of life (IBS-QOL) scores, with greater improvement compared to the control group (Zhao et al., 2024) suggesting that acupuncture not only relieves somatic symptoms but also enhances patients’ work and social functioning.

Conventional IBS medications are often associated with side effects. For example, antidepressants may cause dry mouth and drowsiness, while some intestinal drugs can lead to other gastrointestinal discomforts. Some patients are forced to discontinue medication due to side effects, while others—due to personal health beliefs, comorbidities, pregnancy preparation, or other reasons—tend to avoid long-term use of chemical drugs. For these patients, the non-pharmacological nature and minimal side effects of acupuncture make it an attractive alternative (Oka et al., 2020; Shi et al., 2022).

IBS is classified into four subtypes based on predominant symptoms: diarrhea-predominant (IBS-D), constipation-predominant (IBS-C), mixed (IBS-M), and unclassified (IBS-U). Current evidence suggests that the response to acupuncture may vary across subtypes, with IBS-D patients being the most well-documented beneficiaries. The vast majority of high-quality randomized controlled trials on acupuncture for IBS have focused on patients with IBS-D (Wang et al., 2024; Yang et al., 2025). Evidence on the long-term effects of acupuncture—such as sustained symptom improvement for 12 weeks, 18 weeks, or even 24 weeks—also comes primarily from the IBS-D population. Therefore, for patients with IBS-D, acupuncture is a strongly recommended treatment option supported by high-level evidence.

Patients with IBS often suffer from both gastrointestinal symptoms and psychological distress; anxiety and depression are the most common comorbidities of IBS. For these patients, acupuncture offers a unique “killing two birds with one stone” therapeutic advantage. Thus, for IBS patients with significant anxiety and/or depression, acupuncture is not only appropriate but also an particularly ideal treatment choice.

Some patients naturally have a preference for holistic medicine. They seek not merely symptom suppression but overall balance and harmony of bodily systems. They may be skeptical of the “antagonistic” treatment model of Western medicine and instead favor “regulatory” therapies like acupuncture, which aim to stimulate the body’s self-healing capacity.

Despite the generally positive evidence, there remains academic debate as to whether acupuncture for IBS produces only a significant placebo effect and whether its efficacy truly surpasses that of sham acupuncture (placebo acupuncture). One study clearly indicated that both the acupuncture and sham acupuncture groups showed significant improvement in global quality of life at the end of treatment, with no statistically significant difference between the two groups, suggesting that non-specific effects may play a dominant role (Shanshal et al., 2023).

Therefore, sham acupuncture in research settings is not a completely inert placebo control. While this nuance should be acknowledged when evaluating the absolute efficacy of acupuncture, it does not diminish its clinical value as a low-risk, patient-centered treatment option.

### 5.5 Limitations and future directions of current clinical research

Current clinical research has provided preliminary evidence for acupuncture in treating IBS by modulating the gut microbiota. However, several early studies have methodological limitations: relatively small sample sizes, insufficient rigor in randomization and allocation concealment, inadequate blinding, and suboptimal control group designs (e.g., blank controls rather than sham acupuncture) (Wang X. et al., 2022; Yin et al., 2022). These methodological shortcomings may affect the robustness of study conclusions and may partly explain the inconsistent findings across studies, such as those regarding changes in alpha diversity.

Secondly, acupuncture protocols in existing studies are highly heterogeneous, varying in acupoint combinations, stimulation parameters (manual vs. electroacupuncture, frequency, waveform, intensity), treatment frequency, and duration (Lam et al., 2025; Zhou J. et al., 2025). Although acupoints like ST25, ST36, and ST37 are frequently used (Abdullah et al., 2025), their combinations differ across studies. This heterogeneity hampers direct comparison, meta-analysis, and the development of standardized clinical pathways. Furthermore, there is a lack of comparative studies directly examining the differential effects of various acupuncture techniques (e.g., manual acupuncture vs. electroacupuncture) on gut microbiota modulation (Wang et al., 2021; Tian et al., 2023).

Most studies lack long-term follow-up, leaving critical questions unanswered: how long do effects persist post-treatment? Does microbiota remain stable or return to baseline? Does symptom recurrence correlate with renewed dysbiosis? (Botschuijver et al., 2019) Existing evidence is primarily based on correlation analyses; although these reveal strong associations between microbiota changes and symptom improvement, establishing causality requires more rigorous experimental validation. Additionally, few clinical trials have directly compared acupuncture head-to-head with first-line standard pharmacological treatments for IBS (e.g., antispasmodics, laxatives, or antidepressants) specifically regarding gut microbiota modulation (Botschuijver et al., 2019; Chen et al., 2021c; Qin et al., 2022; Xing et al., 2025a). Such comparative studies are valuable for defining acupuncture’s position within the overall IBS treatment strategy—whether as an alternative, adjunctive, or integrative therapy (Chen et al., 2024; Xing et al., 2025b).

Combination therapies with probiotics/prebiotics warrant exploration: by optimizing the intestinal microenvironment, acupuncture may create favorable conditions for exogenous probiotic colonization, producing synergistic effects (Long et al., 2006; Botschuijver et al., 2019; Goodoory et al., 2023).

In-depth investigation of gut-brain axis mechanisms remains crucial: future research should elucidate how acupuncture modulates signaling molecules (serotonin, GABA, SCFAs) to influence microbiota, and how microbiota changes feedback to regulate these pathways (Ding et al., 2020; Yang et al., 2023). Addressing these questions requires integrated application of multiple techniques—including neuroimaging, electrophysiology, and molecular biology—in bidirectional validation studies involving both animal models and human trials.

## 6 Discussion and future perspectives

Acupuncture’s therapeutic mechanisms in IBS may not target a single component but rather initiate a cascade reaction centered on gut microbiota remodeling, systematically modulating the dysregulated MGB axis network and ultimately achieving comprehensive improvement of the complex symptom cluster. This process can be conceptualized as evolving from a localized intervention (acupuncture stimulation) into systemic physiological rebalancing (Figure 1). In contrast to previous studies that often regarded post-acupuncture microbiota changes as epiphenomena, this review posits that such changes may serve as a critical mediator within the acupuncture effect network. Evidence indicates that acupuncture’s modulation is primarily manifested in two aspects: correcting overall dysbiotic structure and regulating specific key bacterial genera.

Structurally, acupuncture reverses dysbiosis features in IBS. In animal models, acupuncture consistently promotes alpha diversity recovery toward healthy levels (Ding et al., 2020; Lam et al., 2025), associated with a more stable, resilient microecosystem. In clinical settings, however, this effect is more variable and may depend on baseline characteristics, disease subtypes, and treatment protocols.

Cross-disciplinary research has established alpha diversity as a core indicator of stability and resilience: decreased diversity in pediatric cohorts correlates with prolonged recovery (Mortensen et al., 2018); low diversity in porcine models characterizes low-resilience phenotypes (Mancin et al., 2024). Thus, acupuncture-induced diversity enhancement helps rebuild a perturbation-resistant microecosystem. Notably, among treatment responders, diversity improvement may be more pronounced, with differences observable as early as week 4, suggesting that microbial diversity improvement may be an early biological event underlying clinical efficacy.

Acupuncture also exerts bidirectional regulation on key functional genera. Extensive animal and clinical studies show that it increases beneficial bacteria, particularly SCFA-producing genera (Xu et al., 2024). For example, *Lactobacillus* and *Bifidobacterium* increase post-treatmen (Xu et al., 2024). Some studies further indicate that acupuncture specifically increases *Faecalibacterium prausnitzii*, a butyrate-producing bacterium with anti-inflammatory properties; butyrate is key for maintaining intestinal barrier integrity and immune homeostasis. Concurrently, the abundance of certain potential pathogens associated with inflammation or intestinal dysfunction, such as Enterobacter and Enterococcus, tends to decrease after acupuncture intervention (Toscano et al., 2017; Vitellio et al., 2019).

This selective enrichment/suppression pattern is not unique to acupuncture. In IBS, probiotic and low-FODMAP diet interventions also induce beneficial bacteria proliferation and pathogen suppression (Long et al., 2006; Goodoory et al., 2023; Naseri et al., 2021); in metabolic diseases, dietary fiber-mediated remodeling similarly optimizes community structure (Ding et al., 2020; Lancaster et al., 2022). This suggests that beneficial enrichment with pathogen attenuation represents a universal ecological response pattern as the microbiota approaches a healthy steady state. Acupuncture’s unique value lies in its ability to trigger this ecological optimization by activating endogenous neuro-immune pathways, without relying on exogenous probiotics or dietary substrates, thereby achieving systemic gut microbiota remodeling.

At the functional level, microbiota composition changes reflect systemic metabolic shifts. Functional prediction analyses show that acupuncture upregulates pathways related to SCFA metabolism and amino acid biosynthesis, while suppressing pro-inflammatory pathways like LPS biosynthesis. This indicates that acupuncture modulates not only the taxonomic composition but also the metabolic capacity of the gut microbiota. This functional optimization shifts the intestinal metabolite profile toward a healthier state, establishing a metabolic foundation for efficient MGB axis signaling.

Once structural remodeling is achieved, bioactive signals are transmitted via the MGB axis’s neural, endocrine, and immune pathways, exerting systemic CNS effects. By remodeling the microbiota, acupuncture modulates gut-brain neural communication, a process critical for alleviating visceral hypersensitivity—a core feature of IBS.

Acupuncture modulates neural pathways through a dual mechanism: it enhances afferent vagal signaling indirectly by enriching SCFA-producing bacteria, while directly activating efferent vagal pathways to initiate the cholinergic anti-inflammatory pathway. This distinguishes acupuncture from single-target strategies like probiotic supplementation or neuromodulation devices alone. Crucially, neuroimaging confirms that acupuncture also directly regulates excitability and connectivity in brain regions such as the anterior cingulate cortex and insula (Chen et al., 2021b; Xu et al., 2023).

Acupuncture’s restoration of the intestinal barrier and immune homeostasis represents another core mechanism for attenuating peripheral nociceptive input. By enriching butyrate-producing bacteria, upregulating tight junction expression, and inhibiting overactivation of the TLR4/NF-κB and NLRP3 inflammatory pathways, acupuncture effectively reduces intestinal permeability and the risk of LPS translocation. More importantly, short-chain fatty acids—particularly butyrate—shift the intestinal immune microenvironment from a “pro-inflammatory” toward an “anti-inflammatory” state by promoting Treg differentiation and inhibiting Th1/Th17 responses. This “barrier repair-immune resetting” cascade not only blocks the aberrant peripheral signals driving visceral hypersensitivity at their source but may also contribute to the improvement of non-gastrointestinal symptoms in IBS patients, such as fatigue and brain fog.

These three major pathways—neural, endocrine, and immune—do not operate in isolation but constitute a highly integrated, interactive regulatory network. The realization of acupuncture’s therapeutic effects may precisely involve activating and coordinating this network, thereby producing synergistic and amplified overall effects. For example, overactivation of the HPA axis, an endocrine pathway abnormality, leads to increased cortisol release, which can directly increase intestinal permeability and impair immune barrier function. Concurrently, stress signals can influence intestinal motility and immune cell activity via neural pathways such as the vagus nerve. Conversely, cytokines produced by intestinal low-grade inflammation (an immune pathway) can cross the blood-brain barrier or influence central function via neural routes, contributing to anxiety and hyperalgesia. The gut microbiota and its metabolites (e.g., SCFAs) occupy a central position within this network, capable of directly influencing distant organs (such as the brain) via the circulation, while also modulating the local enteric nervous system and intestinal immune system; their influence permeates all pathways.

The uniqueness of acupuncture lies in its mechanism, which does not target a single node but rather uses the gut microbiota as a hub to drive the synergy of neural, immune, and endocrine pathways, forming an interactive and amplified beneficial network effect. Improvement in microbiota structure first helps stabilize the local intestinal environment (including barrier repair and inflammation resolution). This stabilized local signal is then transmitted upward via neural and humoral pathways, thereby helping to correct central hyper-responsiveness to stress and pain sensitization. This “point-to-surface” networked regulatory pattern aligns closely with the holistic perspective of traditional Chinese medicine theory and provides a mechanistic understanding for explaining how acupuncture can simultaneously improve both gastrointestinal symptoms and comorbid issues such as mood disturbances and sleep disorders in IBS patients.

This review has summarized acupuncture’s regulation of the MGB axis into three major pathways—neural, endocrine, and immune—elucidating key functional nodes within each and their potential interactions.

An ideal study would integrate multidimensional data—gut metagenomics, host/microbial metabolomics (feces and blood), colonic transcriptomics/proteomics, and central neuroimaging/neurotransmitter data—from the same cohort, using advanced bioinformatics for cross-omics correlation and causal inference. However, most existing studies are limited to “single-omics” analyses. Although research has observed that acupuncture can influence the gut microbiota and also modulate central nervous system activity, how specific metabolites produced by the microbiota exert their effects through precise pathways remains unclear.

Furthermore, do different acupuncture parameters (acupoint combinations, stimulation frequency/intensity, needle retention time) differentially modulate the gut microbiota? This dose-effect and acupoint-specificity question is crucial for advancing acupuncture standardization, yet current explorations are extremely limited. For example, existing research is insufficient to systematically explain, from a microbiome perspective, why specific acupoints such as “Zusanli” (ST36) and “Tianshu” (ST25) are therapeutically effective; the intrinsic connection between these acupoints and gut microbiota regulation requires further in-depth investigation.

Current published clinical trials exhibit significant heterogeneity in study design, including variations in diagnostic criteria, acupoint selection protocols, control group settings (e.g., types of sham or placebo acupuncture), and treatment courses. This substantial heterogeneity poses considerable challenges for conducting high-quality meta-analyses, thereby affecting the overall evaluation and consensus formation regarding acupuncture’s therapeutic efficacy and mechanisms of action.

Notably, individual differences in gut microbiota may lead to variable acupuncture responses. However, current studies employ uniform protocols, potentially overlooking differential responses based on “enterotypes” or baseline microbiota, thus failing to achieve personalized treatment. A major bottleneck in current acupuncture-microbiome research is that the vast majority of studies do not make raw 16S rRNA sequencing data and matched clinical data publicly available. This lack of data sharing severely constrains independent validation and integrative analysis, hindering the effective accumulation of knowledge in the field.

In summary, this review systematically examines acupuncture’s mechanisms in treating IBS via the MGB axis, focusing on microbiota modulation and its multi-level effects via neural, endocrine, and immune pathways. With the gut microbiota as a central hub, acupuncture achieves integrative regulation from localized intervention to systemic homeostasis reconstruction. This perspective offers a novel theoretical framework for understanding the scientific basis of acupuncture in treating IBS.
